# Comparison of Ketamine/Diazepam and Tiletamine/Zolazepam Combinations for Anaesthesia Induction in Horses Undergoing Partial Intravenous Anaesthesia (PIVA): A Retrospective Clinical Study

**DOI:** 10.3390/vetsci11120612

**Published:** 2024-11-30

**Authors:** Carlotta Lambertini, Elena Boanini, Isabelle Casalini, Francesca Spaccini, Riccardo Rinnovati, Noemi Romagnoli

**Affiliations:** Department of Veterinary Medical Sciences, University of Bologna, 40126 Bologna, Italy; elena.boanini2@unibo.it (E.B.); isabelle.casalini2@unibo.it (I.C.); francesca.spaccini2@unibo.it (F.S.); riccardo.rinnovati2@unibo.it (R.R.); noemi.romagnoli@unibo.it (N.R.)

**Keywords:** anaesthesia, equine, ketamine, tiletamine, zolazepam

## Abstract

The aim of this retrospective clinical study was to compare the combinations of ketamine/diazepam (KD group) and tiletamine/zolazepam (TZ group) for the induction of general anaesthesia in horses undergoing elective surgery. The data from the clinical and the anaesthetic records of 138 horses from 2021 to 2023 were evaluated, and the horses were divided in two groups: KD (*n* = 60) and TZ (*n* = 72). The horses were premedicated with romifidine and methadone IV; anaesthesia was induced with ketamine/diazepam for the KD group and tiletamine/zolazepam for the TZ group and was maintained with isoflurane and a constant rate infusion of romifidine. The data encompassed sex and neuter status, age, breed, weight, American Society of Anaesthesiologists physical status, type of surgical procedure performed under anaesthesia, induction time, induction score, surgery time, recovery time, and the recovery score using a descriptive scale. Baseline heart rate (HR), intraoperative HR, baseline respiratory rate (*f*R), intraoperative *f*R, mean arterial pressure (MAP), oxygen saturation (SpO_2_), and fraction of expired isoflurane (F_E_’Iso) were also recorded. The induction time was significantly longer (*p* = 0.004) in the TZ group (60 (40–120)) as compared to the KD group (50 (30–120)). Recovery time was also significantly longer (*p* ≤ 0.001) in the TZ group (46.5 (15–125)) as compared to the KD group (30 (5–105)). These findings suggested that, in adult horses undergoing elective surgery, TZ could be considered a valid alternative to KD for the induction of general anaesthesia. Additional experimental studies comparing the two induction regimens and their pharmacokinetic and pharmacodynamic characteristics are needed.

## Simple Summary

The induction of general anaesthesia in horses is commonly achieved with a dissociative anaesthetic, usually ketamine, in combination with a benzodiazepine (diazepam or midazolam). Tiletamine is another dissociative anaesthetic commonly used in several animal species, which is marketed in combination with the benzodiazepine zolazepam. Previous studies have already compared ketamine and tiletamine for anaesthesia induction in horses, primarily in the context of TIVA, halothane-based inhalant anaesthesia, or intramuscular general anaesthesia. However, their use has not been explored within a partial intravenous anaesthesia (PIVA) protocol. The purpose of this study was to compare the effects of the ketamine/diazepam (KD) or tiletamine/zolazepam (TZ) combinations used for the induction of general anaesthesia in horses undergoing PIVA (isoflurane-romifidine anaesthesia) for elective surgical procedures. The data collected included induction and recovery times, induction and recovery score, isoflurane requirement, and intraoperative cardiorespiratory parameters. The results showed that the anaesthetic effects of the TZ combination were similar to the KD group in terms of induction and recovery qualities, isoflurane requirements, and hemodynamic and respiratory effects. However, the administration of the TZ combination accounted for longer induction and recovery times.

## 1. Introduction

Anaesthetic induction in horses should ideally provide a rapid and uneventful loss of consciousness and recumbency to avoid injury to the personnel or the animal [1]. Dissociative anaesthetics, such as ketamine and tiletamine, are widely used as induction agents in this species, whether undergoing inhalational anaesthesia, partial intravenous anaesthesia (PIVA) in the clinical setting, or total intravenous anaesthesia (TIVA) under field conditions. Dissociative anaesthetics provide a “dissociative anaesthesia” characterised by catalepsy, immobility, increased muscle tone, tremors, nystagmus, and the persistence of palpebral and swallowing reflexes with or without loss of consciousness [1].

Ketamine with benzodiazepine (diazepam or midazolam) is the combination most commonly used for anaesthesia induction in horses [2]. Ketamine is a phencyclidine derivative acting as a non-competitive N-methyl-d-aspartate (NMDA) antagonist, and as demonstrated in animal models or in vitro studies, also acts upon the opioid, monoaminergic, and muscarinic receptors [3,4,5]. When ketamine is administered as IV, the peak plasma concentration occurs within one minute, with the maximal effect occurring within 1–2 min [1]. The duration of the anaesthesia is short, lasting 15 to 20 min [6,7].

Similar to ketamine, tiletamine is the other dissociative anaesthetic used in equine practice. The commercially available preparation consists of tiletamine combined with zolazepam, a benzodiazepine derivative licensed for use in animals but only in combination with tiletamine, with which it is associated for its myorelaxant effect [8]. Zolazepam is characterised by an analogous pharmacological effect to diazepam, even if it is four times more powerful [1,9]. Similarly, tiletamine, when compared with ketamine, is two to four times more powerful [1].

After tiletamine/zolazepam (TZ) administration in horses, induction occurs within 45 to 120 s [6,10,11]. Overall, the induction quality after TZ administration has been described as being smooth, with excellent muscle relaxation resulting from the benzodiazepine association and the myorelaxant effect of an alpha_2_ agonist, which is usually administered in horses for premedication [6,11], though stiffness of the neck or of the limb is sometimes observed [7]. As compared with ketamine, when used under field conditions, the anaesthesia provided by TZ lasts longer, at 52 to 97 min [10]. A drawback of TZ induction is the recovery quality, which has been described as being poor by some authors [6].

Though KD and TZ combinations present a similar cost (approximately EUR 35 to 40), TZ carries some advantages over ketamine for veterinary practice. In fact, even if the TZ is not without human abuse risk [12], its consumption is less widespread compared with ketamine; this is why it is not yet classified as a controlled substance in some countries. Another potential advantage consists instead of its lower volume of injection when compared with ketamine, thereby facilitating administration.

Ketamine and TZ have been described and compared in horses as induction drugs before TIVA [6,7,13,14] or halothane-based inhalant anaesthesia [15], but also as part of total intramuscular anaesthesia protocols [16]. Based on the authors’ knowledge, these combinations have never been investigated for the induction of PIVA. The purpose of this study was to compare the ketamine/diazepam (KD) combination with tiletamine/zolazepam (TZ) combination for the induction of general anaesthesia in horses undergoing isoflurane and romifidine-based PIVA for elective surgical procedures in a clinical setting. The induction and recovery qualities, cardiorespiratory effects, and isoflurane requirement were retrospectively evaluated.

## 2. Materials and Methods

The study was conceived as a retrospective clinical study. The clinical and anaesthetic records of horses undergoing elective surgery at the Veterinary Teaching Hospital of the University of Bologna, Italy, between 2021 and 2023 were reviewed using an electronic patient database (software Fenice, Zakasoft, Vers. 05.56.O520). Written or oral informed owner consent was obtained before submitting each horse to the anaesthetic procedure. The study was performed in accordance with the Ethics Committee of the University of Bologna, conforming to the Dl.vo n. 26/2014 of the Italian government.

The inclusion criteria were as follows: horses over 1 year of age undergoing scheduled procedures having an American Society of Anaesthesiologists’ (ASA) physical status of I to II and adhering to the anaesthetic protocol described below. When the anaesthetic records were reviewed, the horses were then divided into two groups based on the combination of drugs they had received for induction of anaesthesia: a ketamine and diazepam combination for the KD group and tiletamine and zolazepam for the TZ group.

For the aim of this retrospective evaluation, the data recorded in the digital or in the paper records at the time of the surgical procedures were then transcribed into an Excel file (Microsoft Excel for Mac; version 16.87), dividing the horses into the two groups (KD or TZ) depending on the anaesthetic combination used for induction. Also, for statistical evaluation, the surgical procedures the horses underwent were classified into orthopaedic, urogenital, or miscellaneous (soft tissue surgeries, e.g., tumour exeresis) procedures.

The data recorded included sex and neuter status, age, breed, weight, ASA physical status, type of surgical procedure performed under anaesthesia, sedation score, time of induction, quality of induction, surgery time, recovery time, and recovery score.

Standard operating procedures were applied to all the horses included in the study, as follows. After clinical examination, the baseline heart rate (HR) (evaluated by pulse palpation of the maxillary artery and cardiac auscultation) and the respiratory rate (*f*R) (evaluated by visual inspection of the thorax) were recorded. Thereafter, a 14 G catheter (Intranule, VYGON, Paris, France) was inserted into the jugular vein after aseptic preparation of the area.

All the horses were sedated with intramuscular (IM) acepromazine (0.03 mg/kg; Prequillan; Fatro, Italy), intravenous (IV) romifidine (0.08–0.1 mg/kg; Sedivet; Boehringer Ingelheim, Belgium), and IV methadone (0.05 mg/kg; Semfortan; Dechra Pharmaceutics, Northwich, UK). The horses were then moved into a padded box, the sedation score was evaluated using a scale (0–3) modified from Kloppel and Leece [17] (Table A1), and the induction drugs were administered. The anaesthesia was induced with the aid of a gate by two operators: one controlling the head of the horse, and the anaesthetist controlling the gate. General anaesthesia was induced, depending on the choice of the anaesthetist, with an IV combination of diazepam (0.05 mg/kg IV; Ziapam; Ecuphar, Oostkamp, Belgium) and ketamine (2.5 mg/kg IV; Nimatek; Dechra Pharmaceuticals, Northwich, UK) in the KD group or TZ at a dose of 1 mg/kg IV in the TZ group (Zoletil 100; Virbac, Milano, Italy). The same anaesthetist also calculated the time of induction (time interval between the end of the injection of the induction dose and moment at which the horse reached the ground) with the aid of a digital clock and the induction score, using a descriptive scale (0–3) previously described by Marntell et al. [18] (Table A2).

Once the horse was recumbent, the trachea was intubated, and the animal was placed on a surgical table and connected to a large-animal circle system. The anaesthesia was maintained using isoflurane delivered in a mixture of air and oxygen (1:1 ratio), in combination with a romifidine infusion (0.04 mg/kg/h). The horses were kept on spontaneous ventilation and, only if they developed an apneustic breathing pattern, mechanical ventilation was applied using an Equine ventilator (ALPHA 400 MKII, Minerve, France). For the aim of the statistical evaluation, the number of horses receiving mechanical ventilation in the two groups was not taken into consideration. The level of anaesthesia was assessed and adjusted by the anaesthetist based on experience and clinical circumstances and was considered unrelated to the objectives of the study. Once the horses were in the operating theatre, the recumbency was decided based on the surgical procedure, a complete cardiovascular monitoring was applied using a multiparametric monitor (Datex-Ohmeda S/5, Datex Omeda S.p.A, Milan, Italy), and measurement of the heart rate (HR) with an electrocardiogram, (*f*R), fraction of expired CO_2_ (F_E_’CO_2_) and fraction of expired isoflurane (F_E_’Iso) % using a side stream calibrated gas analyser, haemoglobin oxygen saturation using a pulse oximeter (SpO_2_), and body temperature were evaluated. An arterial catheter was placed in the transverse facial artery or in the facial artery for monitoring of the invasive systolic (SAP), diastolic (DAP), and mean arterial (MAP) pressures. Eventual arrhythmias were also recorded in the anaesthetic record and, if present, were taken into consideration in the analysis. In addition, upon application of the surgical stimulation in order to maintain a stable surgical anaesthetic plane, the anaesthetist assessed the anaesthetic depth based on vital signs (nystagmus, palpebral reflex, position of the eye globe, spontaneous movements) at five-minute intervals. The anaesthetic depth was corrected as needed by increasing or decreasing the end-tidal isoflurane concentration. In the case of movement of the limbs or of the head, thiopental 1 mg/kg (Pentothal sodium 1 gr, MSD Animal Health S.r.l., Segrate, MI, Italy) was administered IV. For statistical purposes, the number of thiopental boluses was taken into consideration. Hypotension was defined as a MAP < 70 mmHg and was treated with IV dobutamine (Dobutamina, Bioindustria L.I.M., Novi Ligure, Italy) starting from 0.0005–0.0015 mg/kg/min until normalisation of the MAP. In the case of inadequate analgesia, considered to be a 20% increase in HR and MAP as compared with the value recorded before the beginning of the surgery, lidocaine was administered by IV at 0.05 mg/kg/min. Ringer Lactated solution (B.Braun Vet Care Ringer Lattato Hartman, B.Braun S.p.A, Milan, Italy) was administered intravenously throughout the anaesthesia at 10 mL/kg/h. All the horses received flunixin meglumine (Meglufen 50 mg/mL, IZO S.r.l, Brescia, Italy) 1.1 mg/kg IV intraoperatively.

At the conclusion of the surgery, isoflurane was discontinued (end of anaesthesia); the horses were then placed in a padded box in lateral recumbency, and romifidine 0.02 mg/kg was administered IV. The endotracheal tube was removed when the swallowing reflex was observed or within 15 min after the end of the anaesthesia After extubation, the horses were left in the box to recover and were monitored until they achieved a firm standing position, as observed with the aid of a camera (Reolink camera 5 Mb wi-fi, RVaeolink, Hong Kong) connected wirelessly to a computer or to a smartphone. The same anaesthetist evaluated the recovery time (time interval between the extubation and the standing position) and recorded the quality of the recovery on the anaesthetic record by using a descriptive scale (0–5) as previously described by Gonzalo-Marcilla et al. [2] (Table A3).

### Statistical Analysis

Statistical analysis was carried out using statistical computer software (Stata/SE 17.0, StataCorp, TX, USA). For statistical purposes, the mean intraoperative values for each variable were taken into consideration for each horse (HR, *f*R, MAP, F_E_’Iso, and SpO_2_). Single outliers were identified using Microsoft Excel software and were not taken into consideration for the statistical evaluation. Some of the data were missing in the anaesthetic records: when applicable, the number of the horses for which data were available are specified in the results section.

The data were evaluated for normality using a Shapiro–Wilk test. The normally distributed data were compared between the two groups using a Student-*t* test and were reported as mean ± standard deviation (SD). The non-normally distributed data were compared between groups using a Mann–Whitney test and are reported as median and range.

The baseline and intraoperative value for HR and *f*R were compared within each group using a Wilcoxon signed rank test.

The associations between groups and dichotomic data (sex, type of surgery, administration of adjunctive drugs, i.e., lidocaine infusion, dobutamine infusion, or thiopental bolus) were analysed using a Fisher exact test. *p* < 0.05 was considered statistically significant.

## 3. Results

A total of 390 anaesthetic records from horses that underwent general anaesthesia in the period of interest were collected and reviewed. Of these, 67 were younger than 1 year of age, and 43 were classified as ASA ≥ 3. Also, a total of 148 records were further excluded because different anaesthetic protocols were applied (e.g., if the premedication protocol included xylazine, detomidine, butorphanol, or morphine) or the anaesthetic records were unavailable. Finally, a total of 132 horses were included in the study: 60 horses in the KD group and 72 in the TZ group.

The retrospective evaluation highlighted that the demographic data, including the age, sex, and weight of the horses included in the study, did not differ significantly between the two groups; the data are summarised in Table 1. The type of surgical procedure (Table 2) the animals underwent did not differ significantly between the two groups (*p* = 0.65).

The sedation and the induction scores, the induction time, and the duration of the anaesthesia and the surgery are reported in Table 3. The sedation was without complications in all the horses, and the sedation score (evaluated in 44 out of 60 horses in group KD and in 66 out of 72 horses in group TZ) did not differ significantly between the two groups.

The induction time for the horses in the KD group took on average 10 s more as compared to the TZ group, and the difference was statistically significant. Despite this difference, for both groups, the average quality of induction was excellent. In detail, the induction time was recorded in 44 (out of 60) horses in group KD and in 66 (out of 72) horses in group TZ; the quality of induction was recorded in 61 (out of 72) horses in the TZ group. In this latter group, the average induction score was graded as excellent, even though 4 horses had an induction score of 2, and 3 had an induction score of 1. For the horses in the KD group, the average induction, recorded in 43 (out of 60) horses, was graded as excellent, even though 3 horses had an induction score of 1, and 1 horse had an induction score of 0.

After induction, 4 (out of 60) horses in the KD group and 7 (out of 72) horses in the TZ group required a bolus of thiopental (1 mg/kg) before being placed on the operating table, without statistically significant association with the group (*p* = 0.75).

The average *f*R and HR decreased intraoperatively as compared with the baseline. The difference in the *f*R was statistically significant in both groups (*p* < 0.01); for the HR, the difference was statistically significant (*p* = 0.04) only in the KD group. The baseline HR and *f*R, and also the average of the intraoperative values of HR, *f*R, MAP, F_E_’Iso, and SpO_2_ did not differ significantly between the two groups. These results are reported in Table 4. None of the horses developed arrhythmias. The number of horses receiving dobutamine constant rate infusion (CRI) was not significantly associated with the group; it was 50 out of 60 in the KD group and 56 out of 72 in the TZ group (*p* = 0.5).

Intraoperatively, lidocaine infusion (0.05 mg/kg/min), as a rescue analgesia, was provided IV in 14 (out of 60) horses in the KD group and in 15 (out of 72) horses in the TZ group. The number of horses receiving lidocaine did not differ significantly between the two groups (*p* = 0.83).

The recovery score was good and without statistically significant differences between the two groups, while the recovery time was statistically significantly longer in the TZ group as compared with the KD group (Table 3). The recovery score was recorded in all horses in the KD group and in 70 (out of 72) horses in group TZ; the recovery time was recorded in 56 (out of 60) horses in group KD and in 70 (out of 72) horses in group TZ.

## 4. Discussion

The aim of the present study was to compare the anaesthetic effects of KD and TZ combinations on the quality of induction and recovery in horses undergoing isoflurane-romifidine PIVA for an elective procedure.

The results demonstrated that the induction produced by TZ was comparable to that achieved by ketamine/diazepam administration in terms of quality, with both protocols being excellent, even with 10 s longer induction times in group TZ. The induction phase was characterised by good muscle relaxation in both groups. Only a few horses had forward or backward movements with either the KD or the TZ combination.

The lack of difference in the induction quality between KD and TZ was in accordance with previous similar studies in horses [6,7,14]. The comparable quality of induction between KD and TZ was also observed when the two drugs were administered IM in combination with detomidine, providing an overall satisfactory effect [16]. Only Marntell and Nyman [17] reported a superior quality of induction after ketamine administration over TZ.

The good sedation achieved with the premedication protocol administered in the present cohort likely contributed to achieving these results. In fact, sedatives administered prior to induction contribute to reducing awareness and transition to recumbency [1], while inadequate sedation is more likely associated with unsatisfactory induction [6]. In the present study, the horses received acepromazine and romifidine before induction. Acepromazine is a phenothiazine commonly used for premedication in horses, usually in combination with an alpha_2_ agonist, to improve the depth of the sedation before anaesthesia, but also to improve the quality and the speed of induction [19,20].

Different qualities of induction were obtained with TZ depending on the alpha_2_ agonist used for sedation, as previously described by several authors. An overall smooth or excellent quality of induction was reported when TZ was administered for anaesthesia induction in xylazine [6,11,13] and excellent in medetomidine [10] sedated horses; smooth inductions were obtained when TZ was administered in detomidine-sedated horses, even though muscle weakness or attempts to resist recumbency were observed [7]. When romifidine was used for premedication, different observations were reported concerning induction after TZ administration, ranging from smooth up to an abrupt fall of the animal, resulting from a too-rapid muscle relaxation [14,19]. In the horses included in the present study, romifidine was preferred over xylazine or detomidine because, other than a similar sedative effect, it is characterized by a long duration of the sedative effect, with residual sedation lasting up to 200 min when administered at 0.08 mg/kg [21,22]. This duration is longer if compared with sedation provided by xylazine (90 min when administered at 1 mg/kg) and detomidine (up to 110 min when administered at 0.02 mg/kg) [22].

Instead, a significant difference was observed in terms of length of induction between the two groups, being longer for the TZ group (median 60 s) when compared with that obtained after KD administration (median 50 s). Despite the statistically significant difference, the clinical relevance of this difference is debatable even though TZ accounted for a 20% increase of the induction time when compared with KD. Similar induction times of between 75 and 120 s were observed in horses sedated with romifidine before ketamine or TZ induction [14,19]. Other authors have reported shorter induction times, similar to the lower range observed in our cases; in fact, recumbency was achieved in 35.3–37.5 s when TZ (at 1 or 1.65 or 2.2 mg/kg IV) was administered to xylazine-sedated horses [13] and in 35–65 s when TZ 0.7 mg/kg was administered to medetomidine-sedated horses [10]. This difference between premedication protocols was not unexpected. In fact, other authors also observed that horses sedated with romifidine took longer to reach decubitus compared to xylazine and medetomidine [10,13,14,19]. This, as already observed by Kerr and colleagues [23], is likely associated with the greater sedative effect of romifidine, which, providing a state of relaxation and reduced neural activity, slows down the induction phase as compared with other sedatives.

The haemodynamic and respiratory parameters did not differ significantly between the KD or the TZ groups. These results were in accordance with previous studies carried out under field conditions [6]. The HR decreased intraoperatively as compared with baseline values in both groups, even though the difference was statistically significant only for the KD group. The median intraoperative HR was within the ranges reported for anaesthetised horses sedated with romifidine [22], and the decrease in HR could be explained by the administration of romifidine itself [19]. In fact, the latter, by means of its action on the peripheral alpha_2_ adrenoceptors, produces vasoconstriction and reflex bradycardia, which is thereafter sustained by the centrally mediated depression of the sympathetic tone. The administration of either ketamine or TZ for the induction of anaesthesia in horses can return the HR to its baseline values [19]. However, this does not reflect the results of the present study, in which the average intraoperative HR in the KD group was significantly lower when compared with the baseline value; it can be postulated that isoflurane might have blunted its effect. Although irregular rhythms have been reported after ketamine or TZ induction [20], none of these episodes were recorded for the horses included in the present study. Intraoperatively, the average MAP was within normal limits; however, episodes of hypotension were recorded in both groups, and dobutamine was required to maintain a MAP > 70 mmHg without a significant association between the number of horses receiving dobutamine and the induction drugs used. Dobutamine is an inotropic drug that effectively increases the cardiac index in anaesthetised horses; therefore, it is widely used in equine practice to manage anaesthesia-associated hypotension [24,25].

Respiratory depression and hypercapnia are common complications under anaesthesia in spontaneously breathing or mechanically ventilated horses [24,26], and the latter are at a higher risk of developing hypoxaemia as compared with other species [27]. The intraoperative reduction in *f*R observed in this cohort is consistent with the reports of previous studies in horses [10,28]. The F_E_’CO_2_ was not taken into consideration in the present study, since the results might have been biased by the inconsistent application of the mechanical ventilation in the two groups. Hypercapnia (PaCO_2_ > 60 mmHg) does not influence the recovery quality, and moderate to severe hypercapnia might also sustain the cardiopulmonary performance and oxygen delivery in anaesthetised horses [29]. On the contrary, hypoxaemia might negatively affect the quality of recovery and worsen the cardiovascular functions in anaesthetised horses [30]. In the present study, the blood gas evaluation was not considered, as it was not routinely carried out on all the horses included in the study; however, severe hypoxaemia, intended as a SpO_2_ < 90%, corresponding approximately to a PaO_2_ < 60 mmHg, was not detected by the pulsoxymeter evaluation [31]. The F_E_’Iso did not differ between the two groups intraoperatively and was within the minimum alveolar concentration (MAC) of isoflurane previously reported for horses [32].

Concerning the comparison of the recovery characteristic with the two anaesthetic protocols, significant differences were limited to the recovery time; in fact, the horses in the TZ group required more time to recover. Despite this, the quality of recovery did not differ between the two groups, and most of the horses reached the standing position at the first attempt without ataxia. The time for extubation was not considered in the present study, since the horses were in any case extubated 15 min after the end of anaesthesia unless they regained their swallowing reflex sooner. This decision was based on a risk–benefit assessment; indeed, the persistence of the endotracheal tube may not be well tolerated by the horse in the recovery phase and might favour the risk of laryngospasm [33].

Other authors have already described and compared the recovery from anaesthesia in horses after ketamine or TZ; however, a direct comparison is difficult to carry out, as this is the first study in which the two drugs were compared in isoflurane-anaesthetised horses. As regards the time required to stand, in the present study, as also mentioned previously [10,14,34,35,36], the horses induced with TZ required more time to reach recumbency and a standing position when compared with those induced with ketamine. When used for the induction of short-term anaesthesia for castration under field conditions, the effect of TZ lasted longer when compared with ketamine [14]. In fact, according to Bouts and colleagues [14], the mean time for reaching the standing position was 24.6 ± 8.5 min and 48.7 ± 16.8 min, respectively, in ketamine- or tiletamine-anaesthetized horses. According to another study, the anaesthesia provided by TZ lasted even longer, between 56 and 99 min [10]. Also, the duration of the anaesthetic effect after TZ administration has been reported to be dose-dependent, with higher dosages being associated with increased duration of recumbency [11].

Contradictory recovery qualities have been previously described with TZ. Unlike what was indicated by Bouts and colleagues [14], who reported that the recovery from TZ anaesthesia was poor, in this study, the regaining of consciousness after induction with TZ was still good. In the study of Cuvelliez and colleagues, waking up after TZ induction required several attempts before the horse reached a standing position, and the standing position itself was described as precarious; ataxia and hypermetria were also observed [37]. A smooth recovery, with a single attempt to stand [11] or with more attempts, has been reported in detomidine- or xylazine-plus-TZ anaesthetised horses [6,7]. When ketamine or TZ were compared in xylazine-sedated horses, the number of attempts to stand was also significantly higher in the TZ group when compared with the ketamine group [6]. Poor recoveries and some incoordination were also reported after TZ induction in romifidine-sedated horses [14,19]. The quality of the recovery in the present study might have been improved by the administration of romifidine at the end of the anaesthesia. Based on clinical observations and on previous studies, it is highlighted that the quality of post-anaesthesia recovery can be improved by the administration of sedative drugs just before or during this phase [38,39]. By calming the animal, muscle strength and recovery of consciousness can be regained more gradually [40]. Horses not sedated at the end of the anaesthesia show worse recoveries due to early and incoherent attempts to stand [39,40].

A limitation of the present study consists mainly of its retrospective nature [41]. In fact, since the data taken into consideration were not collected for the aim of this study, some were missing, and other data were not collected. Also, another limit of this kind of study is the potential lack of homogeneity and standardization. In fact, also because of the clinical nature of the study, it was not possible to standardise the cases to be included in the study in terms of the duration of anaesthesia, type of surgery, and anaesthesia staff. The involvement of different anaesthetists might have introduced bias in the recording and especially in the evaluations. This latter limitation was partially overcome using standardised scales for the evaluation of the different phases of anaesthesia and the application of standardised protocols. The impossibility of planning the study design is certainly a major limitation of retrospective studies. This also includes the fact that power analysis is more suitable for the evaluation of prospective studies and less suitable for retrospective ones [36]. We cannot therefore exclude the possibility that, in part, the results obtained here can be attributed to the small population included in this research. Despite these limitations, a retrospective analysis is useful for evaluating the association between the protocol used and the anaesthetic effect, especially in a clinical setting where clinical studies encounter some limitations in term of prospective design. The results of the present study might be helpful in designing prospective studies comparing the two induction regimens and their pharmacokinetic and pharmacodynamic characteristics.

## 5. Conclusions

In conclusion, the results of the present study suggest that when TZ is administered to adult horses for the induction of anaesthesia before isoflurane-romifidine PIVA, it provides similar anaesthetic effects when compared to KD in terms of induction and recovery qualities, haemodynamic and respiratory effects, and isoflurane requirements. Also, based on the lack of significant differences, TZ administered at 1 mg/kg can be considered equipotent to the combination of KD administered at the dosages of 2.5 mg/kg and 0.05 mg/kg. Therefore, in adult horses undergoing elective surgery, TZ can be considered a valid alternative to KD for the induction of general anaesthesia maintained with a isoflurane-romifidine combination.

## Figures and Tables

**Table 1:** Demographic data of horses undergoing general anaesthesia and receiving ketamine/diazepam (KD group) or tiletamine/zolazepam (TZ group) for induction. F: females; M: males; CM: castrated males. The data are reported as an absolute number, as median and range (age) or mean ± standard deviation (weight). A *p* value < 0.05 was considered statistically significant. ^a^ *p* value obtained from comparison between the two groups.

Data	KD Group	TZ Group	^a^ *p* Value
Horses (*n* =)	60	72	NA
Age (years)	7 (1–20)	9 (1–22)	0.16
Sex	16 F/31 M/13 CM	23 F/34 M/15 CM	0.801
Weight (kg)	495.4 ± 66.5	481.9 ± 74.9	0.28

**Table 2:** Type of surgical procedure of the horses included in the study. The horses underwent general anaesthesia and received ketamine/diazepam (KD group) or tiletamine/zolazepam (TZ group) for induction. The data are reported as number of horses in each group.

Surgery	KD Group	TZ Group
Miscellaneous	6	10
Orthopaedic	37	48
Orthopaedic + Urogenital	1	1
Urogenital	16	13

**Table 3:** Scores and durations of the anaesthetic phases of horses undergoing general anaesthesia and receiving ketamine/diazepam (KD group) or tiletamine/zolazepam (TZ group) for induction. The data are reported as median and range or mean ± standard deviation. A *p* value < 0.05 was considered statistically significant. * Statistically significant difference as compared with the KD group. ^a^ *p* value obtained from comparison between the two groups.

Evaluations	KD Group	TZ Group	^a^ *p* Value
Sedation score	2 (0–3)	2 (0–3)	0.9
Induction score	3 (0–3)	3 (1–3)	0.97
Induction time (s)	50 (30–120)	60 (40–120) *	0.004
Anaesthesia duration (min)	76 (50–187)	75 (35–223)	0.44
Duration of surgery (min)	40 (15–160)	42.5 (10–180)	0.63
Recovery score	1 (1–4)	1 (1–4)	0.61
Recovery time	30 (5–105)	46.5 (15–125) *	<0.001

**Table 4:** Baseline and intraoperative heart rate (HR), respiratory rate (*f*R), mean arterial pressure (MAP), fraction of expired isoflurane (F_E_’Iso %), and haemoglobin oxygen saturation (SpO_2_) in horses undergoing general anaesthesia and receiving ketamine/diazepam (KD group) or tiletamine/zolazepam (TZ group) for induction. The data are reported as median and range. * Statistically significantly different from baseline (*p* < 0.05); ^§^ statistically significantly different from baseline (*p* < 0.01). ^a^ *p* value obtained from comparison between the two groups.

Parameters	KD Group	TZ Group	^a^ *p* Value
Baseline HR (bpm)	36 (20–60)	36 (24–60)	0.99
Intraoperative HR (bpm)	31.5 (21.5–52) *	35 (20–78)	0.11
Baseline *f*R (bpm)	16 (5–28)	16 (6–28)	0.31
Intraoperative *f*R (bpm)	6.3 (2.5–14) ^§^	7 (3.5–12.5) ^§^	0.46
Intraoperative MAP (mmHg)	81 (60–105)	80 (50–110)	0.08
Intraoperative SpO_2_ (%)	99 (91–100)	99 (92–100)	0.26
Intraoperative F_E_’Iso (%)	1.2 (0.9–1.5)	1.2 (0.9–1.6)	0.89

## Data Availability

The raw data supporting the conclusions of this article will be made available by the authors on request.

## Appendix A

**Table A1:** The quality of sedation was graded using a score modified from Klöppel and Leece (2011) [17].

Grade	Quality of Sedation
0 Unsatisfactory	Unsatisfactory sedation, no or minimal signs of sedation
1 Mild	Moderate sedation with slight dropping of the head
2 Moderate	Moderate sedation with heas drooping mild ataxia
3 Optimal	Marked and deep sedation and ataxia

**Table A2:** The quality of induction was graded using a score previously described by Marntell et al. (2006) [18].

Grade	Quality of Anaesthesia Induction
0 Poor	The horse was induced with considerable movement and/or excitement; the horse may have made subsequent attempts to stand or any other situation which could have resulted in injury.
1 Fair	Recumbency was achieved; the horse fell without relaxation of limbs or with a strong forward or backward movement.
2 Good	Smooth induction, but the horse showed head or limb twitching after induction, or a tendency to walk forward or backward after the induction agent was administered.
3 Excellent	Smooth induction and no muscle twitching. Absent of forward or backward movements.

**Table A3:** The recovery score was graded using a scale proposed by Gonzalo-Marcilla et al. (2021) [2].

Grade	Quality of Recovery
1	One attempt to stand, no ataxia
2	One to two attempts to stand, some ataxia
3	>2 attempts to stand but quiet recovery
4	>2 attempts to stand, excitation
5	Severe excitation, patient injured

## References

[1] Hubbell J.A.E., Muir W.W., Gorenberg E., Hopster K. (2024). A Review of Equine Anesthetic Induction: Are all equine anesthetic inductions “crash” inductions?. J. Equine Vet. Sci..

[2] Gozalo-Marcilla M., Bettschart-Wolfensberger R., Johnston M., Taylor P.M., Redondo J.I. (2021). Data Collection for the Fourth Multicentre Confidential Enquiry into Perioperative Equine Fatalities (CEPEF4) Study: New Technology and Preliminary Results. Animals.

[3] Crisp T., Perrotti J.M., Smith D.L., Stafinsky J.L., Smith D.J. (1991). The local monoaminergic dependency of spinal ketamine. Eur. J. Pharmacol..

[4] Durieux M.E. (1995). Inhibition by ketamine of muscarinic acetylcholine receptor function. Anesth. Analg..

[5] Brockmeyer D.M., Kendig J.J. (1995). Selective effects of ketamine on amino acid-mediated pathways in neonatal rat spinal cord. Br. J. Anaesth..

[6] Matthews N.S., Hartsfield S.M., Cornick J.L., Williams J.D., Beasley A. (1991). A comparison of injectable anesthetic combinations in horses. Vet. Surg..

[7] Wan P.Y., Trim C.M., Mueller P.O. (1992). Xylazine-ketamine and detomidine-tiletamine-zolazepam anesthesia in horses. Vet. Surg..

[8] Lin H.C., Branson K.R., Thurmon J.C., Benson G.J., Tranquilli W.J., Olson W.A., Vähä-Vahe A.T. (1992). Ketamine, Telazol, xylazine and detomidine. A comparative anesthetic drug combinations study in ponies. Acta Vet. Scand..

[9] Lin H.C., Thurmon J.C., Benson G.J., Tranquilli W.J. (1993). Telazol—A review of its pharmacology and use in veterinary medicine. J. Vet. Pharmacol. Ther..

[10] Romagnoli N., Rinnovati R., Lambertini C., Spadari A. (2018). Short-term general anesthesia with tiletamine/zolazepam in horses sedated with medetomidine for castration under field conditions. J. Equine Vet. Sci..

[11] Babbar N., Khandekar G.S., Tripathi S.D., Saini D., Gaikwad S.V., Chauhan S.A. (2023). Clinical Evaluation of Xylazine-Zoletil Anesthesia in Equine under Field Conditions. Indian J. Vet. Sci. Biotechnol..

[12] De la Peña J.B., Cheong J.H. (2016). The abuse liability of the NMDA receptor antagonist-benzodiazepine (tiletamine-zolazepam) combination: Evidence from clinical case reports and preclinical studies. Drug Test. Anal..

[13] Hubbell J.A.E., Bednarski R.M., Muir W.W. (1989). Xylazine and tiletamine-zolazepam anesthesia in horses. Am. J. Vet. Res..

[14] Bouts T., Gasthuys F., Vlaminck L., Van Branteghem L. (2002). Comparison of romifidine-ketamine-midazolam and romifidine-tiletamine-zolazepam total intravenous anaesthesia (TIVA) for clinical anaesthesia in horses. Vet. Anaesth. Analg..

[15] Abrahamsen E.J., Hubbell J.A., Bednarski R.M., Muir W.W., Macioce B.A. (1991). Xylazine and tiletamine-zolazepam for induction of anaesthesia maintained with halothane in 19 horses. Equine Vet. J..

[16] Willette C., Aarnes T.K., Lerche P., Ricco-Pereira C., Ballash G.A., Bednarski R.M. (2021). Evaluation of intramuscular anesthetic protocols in healthy domestic horses. Vet. Anaesth. Analg..

[17] Klöppel H., Leece E.A. (2011). Comparison of ketamine and alfaxalone for induction and maintenance of anaesthesia in ponies undergoing castration. Vet. Anaesth. Analg..

[18] Marntell S., Nyman G., Funkquist P. (2006). Dissociative anaesthesia during field and hospital conditions for castration of colts. Acta Vet. Scand..

[19] Marntell S., Nyman G. (1996). Effects of additional premedication on romifidine and ketamine anaesthesia in horses. Acta Vet. Scand..

[20] Driessen B., Zarucco L., Kalir B., Bertolotti L. (2011). Contemporary use of acepromazine in the anaesthetic management of male horses and ponies: A retrospective study and opinion poll. Equine Vet. J..

[21] England G.C., Clarke K.W., Goossens L. (1992). A comparison of the sedative effects of three alpha 2-adrenoceptor agonists (romifidine, detomidine and xylazine) in the horse. J. Vet. Pharmacol. Ther..

[22] Romagnoli N., Al-Qudah K.M., Armorini S., Lambertini C., Zaghini A., Spadari A., Roncada P. (2017). Pharmacokinetic profile and partitioning in red blood cells of romifidine after single intravenous administration in the horse. Vet. Med. Sci..

[23] Kerr C.L., McDonell W.N., Young S.S. (1996). A comparison of romifidine and xylazine when used with diazepam/ketamine for short duration anesthesia in the horse. Can. Vet. J..

[24] O’Donovan K.A., Aarnes T.K., Hubbell J.A., Parker E.M., Mollenkopf D., Lerche P., Pereira C.H.R., Bini G., Bednarski R.M. (2023). Risk of anesthesia-related complications in draft horses: A retrospective, single-center analysis. Vet. Anaesth. Analg..

[25] Murrell J.C., Grimm K.A., Lamont L.A., Tranquilli W.J., Greene S.A., Robertson S.A. (2015). Adrenergic Agents. Veterinary Anesthesia and Analgesia.

[26] Kieffer P.J., Williams J.M., Shepard M.K., Giguère S., Epstein K.L. (2024). Effect of Hypotension and Dobutamine on Gastrointestinal Microcirculations of Healthy, Anesthetized Horses. Vet. Sci..

[27] Auckburally A., Nyman G. (2017). Review of hypoxaemia in anaesthetized horses: Predisposing factors, consequences and management. Vet. Anaesth. Analg..

[28] Taylor P.M., Bennett R.C., Brearley J.C., Luna S.P., Johnson C.B. (2001). Comparison of detomidine and romifidine as premedicants before ketamine and halothane anesthesia in horses undergoing elective surgery. Am. J. Vet. Res..

[29] Khanna A.K., McDonell W.N., Dyson D.H., Taylor P.M. (1995). Cardiopulmonary effects of hypercapnia during controlled intermittent positive pressure ventilation in the horse. Can. J. Vet. Res..

[30] Meier M., Kazmir-Lysak K., Kälin I., Torgerson P.R., Ringer S.K. (2024). The influence of hypoxaemia, hypotension and hypercapnia (among other factors) on quality of recovery from general anaesthesia in horses. Vet. Anaesth. Analg..

[31] Hopper K., Powell L.L. (2013). Basics of mechanical ventilation for dogs and cats. Vet. Clin. N. Am. Small Anim. Pract..

[32] Dugdale A.H., Taylor P.M. (2016). Equine anaesthesia-associated mortality: Where are we now?. Vet. Anaesth. Analg..

[33] Kim J., Seo B.S. (2013). How to calculate sample size and why. Clin. Orthop. Surg..

[34] Bettschart-Wolfensberger R., Grimm K.A., Lamont L.A., Tranquilli W.J., Greene S.A., Robertson S.A. (2015). Horses. Veterinary Anesthesia and Analgesia.

[35] Muir W.W., Gadawski J.E., Grosenbaugh D.A. (1999). Cardiorespiratory effects of a tiletamine/zolazepam-ketamine-detomidine combination in horses. Am. J. Vet. Res..

[36] Talari K., Goyal M. (2020). Retrospective studies—Utility and caveats. J. R. Coll. Physicians Edinb..

[37] Hubbell J.A., Hinchcliff K.W., Schmall L.M., Muir W.W., Robertson J.T., Sams R.A. (2000). Anesthetic, cardiorespiratory, and metabolic effects of four intravenous anesthetic regimens induced in horses immediately after maximal exercise. Am. J. Vet. Res..

[38] Cuvelliez S., Rosseel G., Blais D., Salmon Y., Troncy E., Lariviere N. (1995). L’anesthésie intraveineuse chez le cheval: Comparaison des combinaisons xylazine-kétamine et xylazine-tilétamine-zolazépam. Can. J. Vet. Res..

[39] Wagner A.E. (2008). Complications in equine anesthesia. Vet. Clin. N. Am. Equine Pract..

[40] Santos M., Fuente M., Garcia-Iturralde P., Herran R., Lopez-Sanroman J., Tendillo F.J. (2003). Effects of alpha-2 adrenoceptor agonists during recovery from isoflurane anaesthesia in horses. Equine Vet. J..

[41] Valverde A., Black B., Cribb N.C., Hathway A., Daw A. (2013). Assessment of unassisted recovery from repeated general isoflurane anesthesia in horses following postanesthetic administration of xylazine or acepromazine or a combination of xylazine and ketamine. Vet. Anaesth. Analg..

